# External validation of machine learning models for predicting prehospital delay in acute ischemic stroke: a retrospective cohort study

**DOI:** 10.3389/fneur.2026.1867290

**Published:** 2026-09-10

**Authors:** Shan Zeng, Aishanjiang Yusufujiang, Hui Dang, Li Zhu, Abudukeyoumu Yasheng, Jingjing Wang, Wei Yan, Huijuan Yu, Gulijanat Mamattursun, Maierpu· Aini, Di Kang, Hongyan Li

**Affiliations:** 1 People’s Hospital of Xinjiang Uygur Autonomous Region, Urumqi, Xinjiang, China; 2 Xinjiang Clinical Research Center for Stroke and Rare Neurological Disease, Urumqi, Xinjiang, China; 3 Kashi Prefecture Second People’s Hospital, Kashi, China; 4 The First People’s Hospital of Kashi Prefecture, Kashi, China

**Keywords:** acute ischemic stroke, external validation, machine learning, prediction model, prehospital delay, TRIPOD

## Abstract

**Background and purpose:**

Prehospital delay remains a major barrier to timely reperfusion therapy in acute ischemic stroke (AIS). We externally validated eight prediction algorithms and examined whether complex machine-learning models added value beyond logistic regression.

**Methods:**

This retrospective cohort study included consecutive patients with AIS from two hospitals in Kashgar, China (January 2019–December 2024). Hospital 1 (*n* = 1,377) was the development cohort and Hospital 2 (*n* = 1,015) the independent validation cohort. Prehospital delay was defined as onset-to-door time >4.5 h. LASSO selection and tuning were restricted to Hospital 1. Sensitivity analyses used all 36 encoded candidate features and repeated all models without SMOTE. Evaluation included discrimination, calibration, post-hoc recalibration, and decision curves.

**Results:**

Delay occurred in 80.9 and 83.2% of the two cohorts. In the prespecified LASSO-plus-SMOTE analysis, XGBoost had the highest validation AUC (0.771; 95% CI, 0.734–0.807), but did not significantly outperform logistic regression (difference, 0.015; 95% CI, −0.003 to 0.034; *p* = 0.107). Full-feature modeling produced no consistent gain. Omitting SMOTE improved calibration and often discrimination; logistic regression without SMOTE achieved AUC 0.774 and Brier score 0.122, versus 0.756 and 0.180 with SMOTE. The original SMOTE-trained logistic model underestimated absolute risk. Rural residence, non-emergency-channel presentation, referral, and non-ambulance transport were associated with absolute risk differences of 15.8, 21.5, 9.2, and 36.3 percentage points in Hospital 2.

**Conclusion:**

Complex algorithms did not show a clear advantage over logistic regression. Because several predictors are available only after arrival, the framework is intended for retrospective health-system profiling and quality-improvement planning, not individual pre-arrival prediction. Universal interventions remain warranted given the high prevalence of delay.

## Introduction

Acute ischemic stroke (AIS) is a leading cause of death and disability worldwide, with time-sensitive treatments such as intravenous thrombolysis and endovascular thrombectomy offering substantial benefits when administered within narrow therapeutic windows (1–3). However, prehospital delay—defined as prolonged onset-to-door time (ODT)—remains a critical barrier to timely reperfusion therapy (4–6).

Understanding factors associated with prehospital delay is important for improving stroke-care delivery (51). Most published prediction models rely on internal validation, which may overestimate performance and does not establish transportability to another clinical setting (7–12, 44, 48). The two-hospital, non-random split by setting used here corresponds to TRIPOD Type 2b under the original TRIPOD classification (13).

Machine-learning algorithms can represent nonlinear associations and interactions, but their added value over simpler methods should be demonstrated rather than assumed (14–17). This is particularly relevant when logistic LASSO is used for feature selection. Moreover, several candidate predictors in this study, including NIHSS and stroke-characteristic variables, are available only after hospital arrival. The intended use is therefore retrospective health-system surveillance, subgroup identification, and quality-improvement planning rather than emergency-dispatch use or individual pre-arrival prediction.

We externally validated eight algorithms using a development cohort from Hospital 1 and an independent validation cohort from Hospital 2. In response to the model-selection concern, we repeated all algorithms with the full candidate feature set and without SMOTE. We also evaluated calibration, *post-hoc* recalibration, decision curves, and subgroup absolute risks for modifiable care-pathway factors.

## Methods

### Study design and setting

This retrospective cohort study was conducted at two tertiary stroke centers in Kashgar Prefecture, Xinjiang, China: People’s Hospital of Kashgar Prefecture (Hospital 1) and First People’s Hospital of Kashgar City (Hospital 2). Both hospitals maintain prospective stroke registries and serve distinct catchment populations. Hospital 1 served as the development cohort and Hospital 2 as the independent validation cohort. The non-random split by hospital corresponds to TRIPOD Type 2b under the original TRIPOD classification (13).

The studies involving human participants were approved by the Clinical Research Ethics Committee of Xinjiang Uygur Autonomous Region People’s Hospital (approval No. KY2224110701) and were conducted in accordance with local legislation and institutional requirements. The participants provided written informed consent to participate in the study. The study was reported in accordance with the Strengthening the Reporting of Observational Studies in Epidemiology (STROBE) statement (47).

### Study population

We included consecutive adult patients (≥18 years) with AIS admitted between January 1, 2019, and December 31, 2024. AIS was diagnosed according to World Health Organization criteria and confirmed by computed tomography or magnetic resonance imaging (18). Exclusion criteria were: (1) wake-up stroke with unknown symptom onset time; (2) missing ODT data; and (3) invalid ODT values (≤0 h). These criteria were applied identically to both hospitals to ensure comparability.

### Outcome definition

The primary outcome was prehospital delay, defined as ODT >4.5 h, corresponding to the extended time window for IV tPA in selected patients (19). ODT was calculated as the time from symptom onset (or last known well time) to hospital arrival, recorded prospectively in the stroke registry.

### Candidate predictors

We identified 36 candidate predictors based on literature review and clinical relevance, covering demographics and clinical history, stroke characteristics, presenting symptoms, prehospital care pathways, and temporal factors. Ethnicity was retained for descriptive reporting but excluded from modeling. Treatment-related variables, including intravenous thrombolysis and thrombectomy, were excluded because they occur after arrival and would create reverse-causality leakage (20, 21).

NIHSS and several stroke-characteristic variables require assessment after hospital arrival. Accordingly, the models were not designed for emergency-dispatch use, real-time pre-arrival triage, or individual prognostication.

### Data preprocessing

All preprocessing parameters were fitted exclusively on Hospital 1 data and then applied to Hospital 2 data to prevent data leakage—a critical methodological safeguard for external validation (49). Missing continuous variables were imputed using median values, and missing categorical variables were imputed using the most frequent category. Continuous variables were standardized using z-score normalization. One-hot encoding was applied to nominal categorical variables with more than two categories.

### Feature selection

We used LASSO logistic regression with 10-fold stratified cross-validation on Hospital 1 to reduce model complexity. One hundred inverse-regularization values from 10^−4^ to 10^2^ were evaluated, and the value maximizing cross-validated AUC was selected. Features with nonzero coefficients were retained for the prespecified analysis (22, 23).

### Model development

Eight algorithms were evaluated: logistic regression, random forest, XGBoost, LightGBM, AdaBoost, support vector classifier, multilayer perceptron, and Gaussian naive Bayes (50). Hyperparameters were selected by grid search using identical five-fold stratified splits in Hospital 1. The complete search grids and selected values are reported in Supplementary Table S2. Random state 42 was used.

The prespecified analysis applied SMOTE (sampling strategy “auto,” five nearest neighbors, random state 42) inside each training fold only. Validation folds and Hospital 2 contained only observed records. After tuning, models were refitted on Hospital 1 and evaluated once in Hospital 2 (24).

### Model evaluation

The primary endpoint was AUC in Hospital 2. Additional measures included Brier score, log loss, accuracy, sensitivity, specificity, PPV, NPV, and F1 score at a probability threshold of 0.50. AUC confidence intervals and paired differences between model predictions were estimated by stratified bootstrap resampling.

Calibration was assessed using calibration-in-the-large, logistic calibration intercept and slope, Brier score, log loss, and calibration curves. Post-hoc logistic and isotonic recalibration were evaluated. Recalibration performance was estimated with 10-fold cross-fitting within Hospital 2, while full-cohort coefficients were reported separately as apparent estimates.

Decision-curve analysis compared each model with reviewing all records and reviewing no records (45). The modeled action was retrospective selection of a patient record for detailed quality-improvement review of EMS access, referral routing, or transport processes. It was not an individual treatment decision. The analysis does not validate aggregation of patient-level scores for subgroup- or service-line resource allocation; such use would require a prespecified aggregation rule and prospective evaluation.

### Sensitivity analyses

To assess whether LASSO selection favored additive models, all eight algorithms were repeated using all 36 non-constant encoded features. Development-cohort permutation importance was calculated for nonlinear models for interpretation. To assess the effect of SMOTE, every LASSO-selected and full-feature model was also trained without SMOTE. Thus, 32 configurations were compared using discrimination, calibration, Brier score, and decision curves.

### Model interpretability

We applied SHAP analysis (25, 43) to the best-performing model using Hospital 2 data to assess external generalizability of feature importance. SHAP values quantify each feature’s contribution to individual predictions based on game-theoretic principles. We generated summary plots showing global feature importance, beeswarm plots showing feature value distributions, separate individual SHAP contribution plots, and dependence plots for the top two features.

### Statistical analysis

Continuous variables were reported as median (IQR) and compared using the Mann–Whitney U test. Categorical variables were reported as number (percentage) and compared using the chi-square or Fisher exact test. Multivariable logistic regression was performed in Hospital 1 (46). Variance inflation factors were calculated with an intercept, and Spearman correlations were examined for key predictors.

In Hospital 2, observed delay risks, absolute risk differences, and risk ratios were estimated for rural residence, non-emergency-channel presentation, referral, and non-ambulance transport, with confidence intervals from 1,000 bootstrap samples. These subgroup estimates are descriptive and not causal effects.

Analyses used Python 3.12 with pandas 3.0.3, NumPy 2.0.2, scikit-learn 1.9.0, statsmodels 0.14.6, imbalanced-learn 0.14.2, XGBoost 3.3.0, LightGBM 4.7.0, and SHAP 0.52.0. All tests were two-sided with *α* = 0.05.

## Results

### Study population

**Figure 1:**
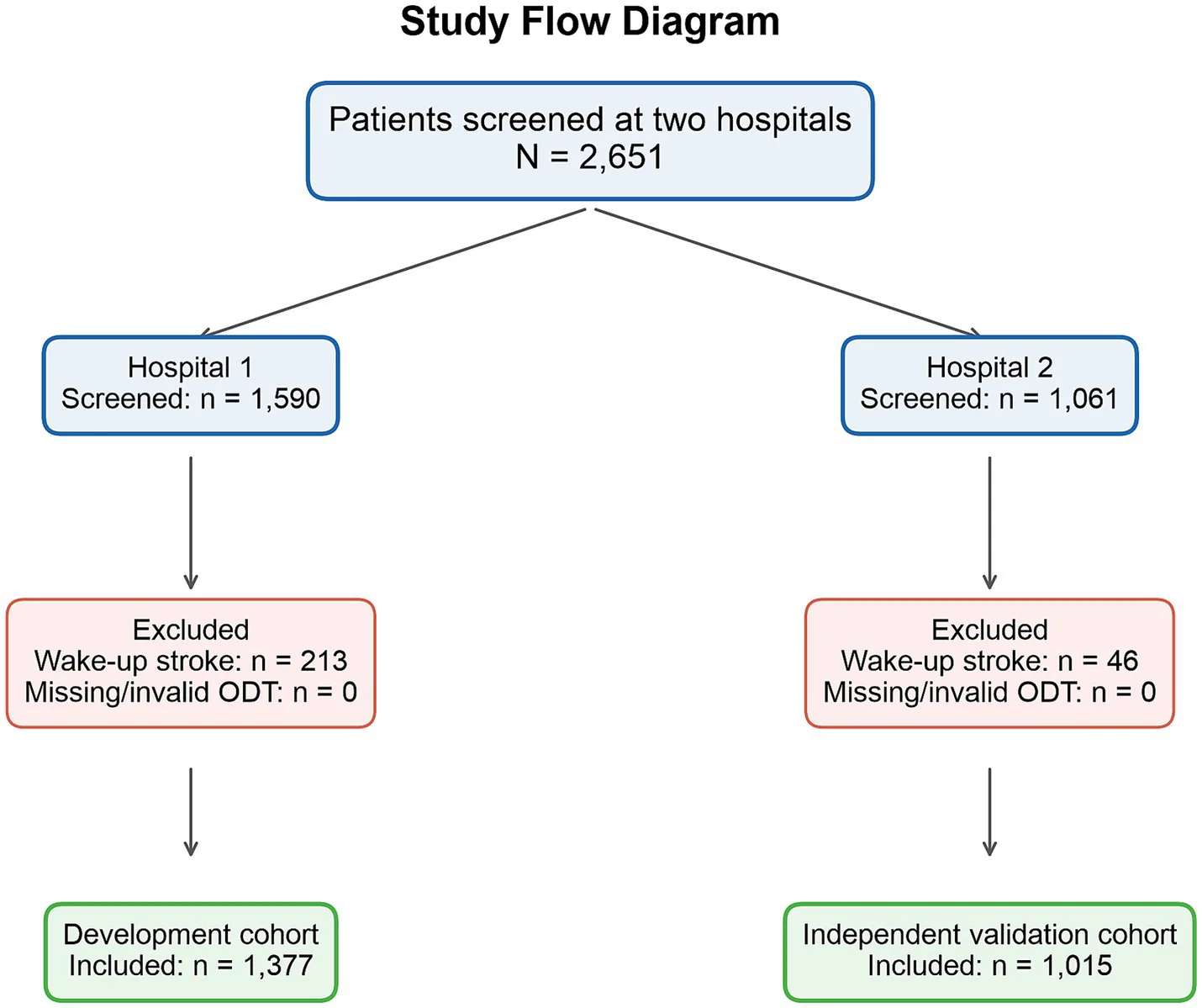
Patient flow diagram.

Between January 2019 and December 2024, 1,590 patients were screened at Hospital 1 and 1,061 at Hospital 2. After excluding 213 and 46 patients with wake-up stroke, respectively, the final cohorts comprised 1,377 Hospital 1 patients and 1,015 Hospital 2 patients (Figure 1). No additional record had missing or invalid ODT after this exclusion.

### Baseline characteristics

Baseline characteristics are summarized in Table 1. Prehospital delay occurred in 1,114 patients (80.9%) at Hospital 1 and 844 (83.2%) at Hospital 2. Median ODT was 16 h (IQR, 6–48) and 24 h (IQR, 8–40), respectively. The higher Hospital 2 median with a narrower IQR reflects greater concentration near 24 h and a heavier upper tail at Hospital 1; medians and IQR widths need not move in parallel. The corrected ODT distribution is shown in Supplementary Figure S1.

**Table 1:** Baseline characteristics of the development and validation cohorts.

Variable	Category	Hospital 1 (Training) (*n* = 1,377)	Hospital 2 (Validation) (*n* = 1,015)	*p* value	Statistical test
Age (years)	—	63.0 (55.0–72.0)	63.0 (54.0–73.0)	0.870	Mann–Whitney U
NIHSS score	—	3.0 (2.0–7.0)	4.0 (2.0–7.0)	0.002	Mann–Whitney U
Onset-to-door time (hours)	—	16.0 (6.0–48.0)	24.0 (8.0–40.0)	0.001	Mann–Whitney U
Number of risk factors	—	1.0 (1.0–2.0)	1.0 (1.0–2.0)	0.014	Mann–Whitney U
Number of symptoms	—	2.0 (1.0–2.0)	2.0 (1.0–2.0)	0.607	Mann–Whitney U
Sex	Male	846 (61.4%)	616 (60.7%)	—	—
	Female	527 (38.3%)	399 (39.3%)	—	—
Age group	<50 years	152 (11.0%)	125 (12.3%)	—	—
	50–59 years	285 (20.7%)	242 (23.8%)	—	—
	60–69 years	343 (24.9%)	239 (23.5%)	—	—
	70–79 years	257 (18.7%)	233 (23.0%)	—	—
	≥80 years	330 (24.0%)	171 (16.8%)	—	—
Educational level	Primary or below	13 (0.9%)	547 (53.9%)	—	—
	Secondary	2 (0.1%)	344 (33.9%)	—	—
	College or above	1 (0.1%)	91 (9.0%)	—	—
	Unknown	1,361 (98.8%)	33 (3.3%)	—	—
Ethnicity	Han	178 (12.9%)	152 (15.0%)	—	—
	Uyghur	1,188 (86.3%)	853 (84.0%)	—	—
	Other	11 (0.8%)	10 (1.0%)	—	—
Occupation	Retired	250 (18.2%)	185 (18.2%)	—	—
	Freelance	141 (10.2%)	46 (4.5%)	—	—
	Farmer	642 (46.6%)	599 (59.0%)	—	—
	Employee	84 (6.1%)	73 (7.2%)	—	—
	Civil servant	25 (1.8%)	19 (1.9%)	—	—
	Other	235 (17.1%)	93 (9.2%)	—	—
Residence	Urban	617 (44.8%)	469 (46.2%)	—	—
	Rural	760 (55.2%)	546 (53.8%)	—	—
Hypertension	No	463 (33.6%)	315 (31.0%)	—	—
	Yes	914 (66.4%)	700 (69.0%)	—	—
Diabetes mellitus	No	1,032 (74.9%)	715 (70.4%)	—	—
	Yes	345 (25.1%)	300 (29.6%)	—	—
Coronary Artery Disease	No	1,099 (79.8%)	744 (73.3%)	—	—
	Yes	278 (20.2%)	271 (26.7%)	—	—
Prior stroke	No	1,143 (83.0%)	879 (86.6%)	—	—
	Yes	234 (17.0%)	136 (13.4%)	—	—
Atrial fibrillation	No	1,329 (96.5%)	993 (97.8%)	—	—
	Yes	48 (3.5%)	22 (2.2%)	—	—
Emergency channel	Emergency department	1,078 (78.3%)	774 (76.3%)	—	—
	Outpatient	291 (21.1%)	226 (22.3%)	—	—
	Unknown	8 (0.6%)	15 (1.5%)	—	—
Transport to hospital	Ambulance (120)	165 (12.0%)	67 (6.6%)	—	—
	Private car	98 (7.1%)	20 (2.0%)	—	—
	Other	8 (0.6%)	4 (0.4%)	—	—
	Unknown	1,106 (80.3%)	924 (91.0%)	—	—
Inter-facility referral	No	877 (63.7%)	789 (77.7%)	—	—
	Yes	500 (36.3%)	226 (22.3%)	—	—
Onset time (night)	1	668 (48.5%)	473 (46.6%)	—	—
	2	708 (51.4%)	540 (53.2%)	—	—
Weekend onset	Weekday	1,017 (73.9%)	717 (70.6%)	—	—
	Weekend	359 (26.1%)	296 (29.2%)	—	—
Season	Spring	435 (31.6%)	242 (23.8%)	—	—
	Summer	336 (24.4%)	245 (24.1%)	—	—
	Autumn	321 (23.3%)	236 (23.3%)	—	—
	Winter	285 (20.7%)	292 (28.8%)	—	—
NIHSS group	Mild (≤5)	837 (60.8%)	637 (62.8%)	—	—
	Moderate (6–9)	166 (12.1%)	151 (14.9%)	—	—
	Moderate–severe (10–16)	180 (13.1%)	110 (10.8%)	—	—
	Severe (17–20)	16 (1.2%)	20 (2.0%)	—	—
	Very severe (>20)	18 (1.3%)	27 (2.7%)	—	—
IV tPA	Yes	147 (10.7%)	174 (17.1%)	—	—
	No	1,230 (89.3%)	841 (82.9%)	—	—
IA thrombectomy	Yes	126 (9.2%)	28 (2.8%)	—	—
	No	1,251 (90.8%)	987 (97.2%)	—	—
Stroke territory	Anterior	869 (63.1%)	553 (54.5%)	—	—
	Posterior	207 (15.0%)	238 (23.4%)	—	—
	Both	271 (19.7%)	151 (14.9%)	—	—
	Unknown	30 (2.2%)	73 (7.2%)	—	—
Large vessel disease	Yes	399 (29.0%)	251 (24.7%)	—	—
	No	832 (60.4%)	623 (61.4%)	—	—
	Unknown	146 (10.6%)	141 (13.9%)	—	—
Motor weakness	No	351 (25.5%)	326 (32.1%)	—	—
	Yes	1,026 (74.5%)	689 (67.9%)	—	—
Slurred speech	No	439 (31.9%)	414 (40.8%)	—	—
	Yes	938 (68.1%)	601 (59.2%)	—	—
Hemianopsia	No	1,315 (95.5%)	969 (95.5%)	—	—
	Yes	62 (4.5%)	46 (4.5%)	—	—
Impaired consciousness	No	1,290 (93.7%)	966 (95.2%)	—	—
	Yes	87 (6.3%)	49 (4.8%)	—	—
Dizziness	No	1,170 (85.0%)	797 (78.5%)	—	—
	Yes	207 (15.0%)	218 (21.5%)	—	—
Impaired sensitivity	No	1,266 (91.9%)	890 (87.7%)	—	—
	Yes	111 (8.1%)	125 (12.3%)	—	—
Facial palsy	No	1,220 (88.6%)	894 (88.1%)	—	—
	Yes	156 (11.3%)	121 (11.9%)		
	Missing	1 (0.1%)	0 (0.0%)		

Percentages are based on available observations. Missingness is reported in Supplementary Table S1A. Educational-level category 4 denotes unknown information and is markedly more frequent in Hospital 1.

### Feature selection

**Figure 2:**
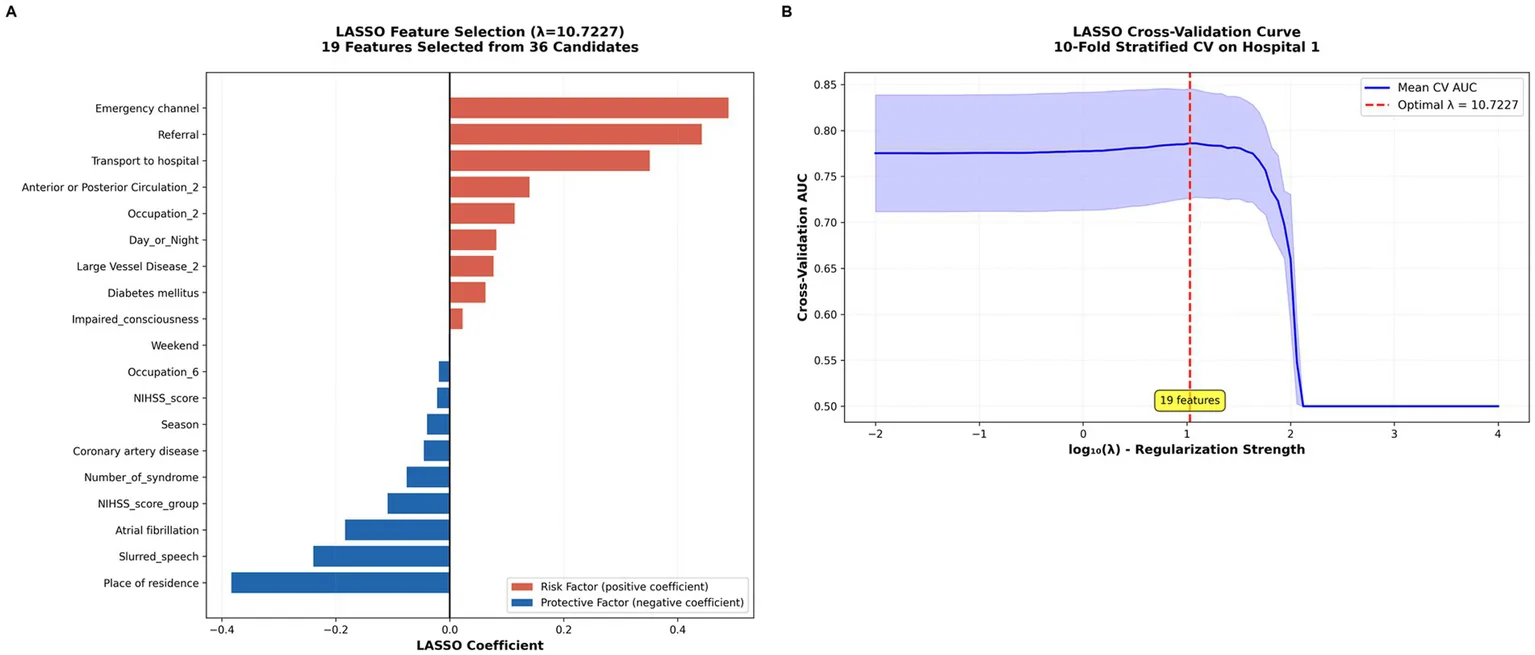
LASSO feature selection. **(A)** Horizontal bar plot of the 19 nonzero LASSO coefficients at the selected regularization value; red and blue bars denote positive and negative coefficients, respectively. **(B)** Cross-validation curve showing mean AUC versus log10 regularization strength (*λ* = 1/C), with the selected *λ* indicated by a vertical dashed line.

LASSO selected 19 of 36 encoded candidate features in Hospital 1 (Figure 2). All preprocessing, feature selection, and tuning were restricted to Hospital 1.

### Multivariable logistic regression

Six factors were independently associated with delay in Hospital 1 (Table 2): non-emergency-channel presentation (aOR, 4.41; 95% CI, 2.53–7.67), absence of atrial fibrillation (aOR, 3.79; 95% CI, 1.95–7.36), non-ambulance transport (aOR, 3.22; 95% CI, 2.20–4.72), referral (aOR, 2.84; 95% CI, 1.94–4.18), absence of slurred speech (aOR, 2.25; 95% CI, 1.57–3.24), and rural residence (aOR, 2.16; 95% CI, 1.57–2.99). VIFs ranged from 1.02 to 1.18, indicating no important multicollinearity.

**Table 2:** Interpretable six-factor logistic regression in Hospital 1.

Predictor	Adjusted OR	95% CI	*p* value
Rural residence	2.16	1.57–2.99	<0.001
Non-emergency channel	4.41	2.53–7.67	<0.001
Inter-facility referral	2.84	1.94–4.18	<0.001
Non-ambulance transport	3.22	2.20–4.72	<0.001
Absence of atrial fibrillation	3.79	1.95–7.36	<0.001
Absence of slurred speech	2.25	1.57–3.24	<0.001

### Machine learning model performance

**Figure 3:**
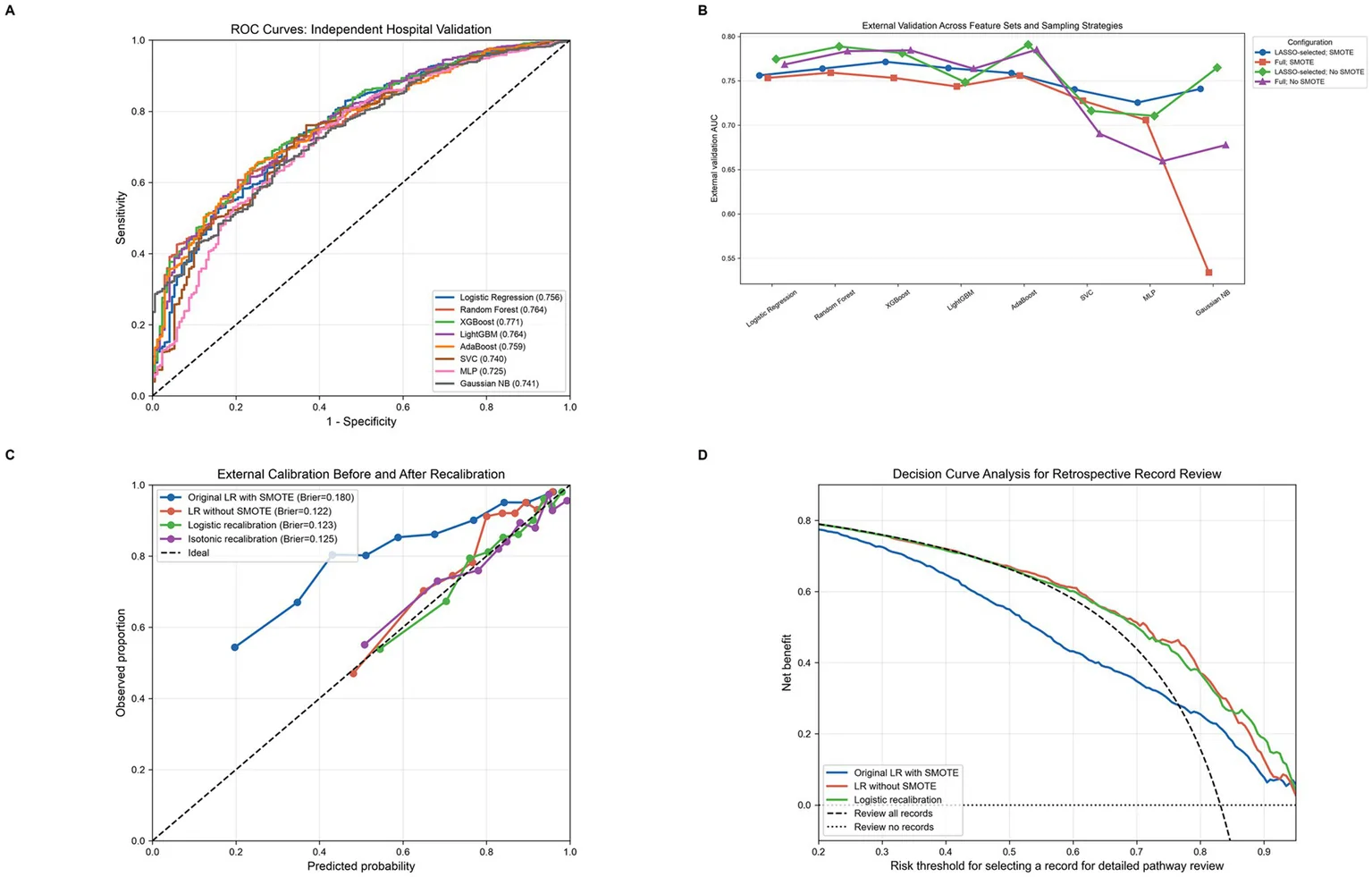
Model performance and sensitivity analyses in Hospital 2. **(A)** ROC curves for the eight prespecified LASSO-plus-SMOTE models. **(B)** External AUCs across LASSO-selected versus full candidate feature sets and SMOTE versus no-SMOTE training. **(C)** External calibration of the original SMOTE-trained logistic model, the no-SMOTE logistic model, and post-hoc logistic and isotonic recalibration. **(D)** Decision-curve analysis for retrospective selection of records for detailed quality-improvement review, compared with reviewing all records and reviewing no records.

In the prespecified LASSO-plus-SMOTE analysis, XGBoost had the highest Hospital 2 AUC (0.771; 95% CI, 0.734–0.807), followed by LightGBM (0.764), random forest (0.764), AdaBoost (0.759), and logistic regression (0.756) (Table 3; Figure 3A). XGBoost sensitivity was 0.764, specificity 0.596, PPV 0.903, NPV 0.339, and Brier score 0.162. The AUC difference between XGBoost and logistic regression was small and not significant (0.015; 95% CI, −0.003 to 0.034; *p* = 0.107). The XGBoost internal out-of-fold AUC was 0.776 (95% CI, 0.746–0.807).

**Table 3:** Model performance across feature-selection and sampling configurations.

Feature set	Sampling	Model	External AUC (95% CI)	Brier	Sensitivity	Specificity	PPV	NPV
LASSO-selected	SMOTE	Logistic Regression	0.756 (0.718–0.797)	0.180	0.732	0.649	0.912	0.329
LASSO-selected	SMOTE	Random Forest	0.764 (0.726–0.800)	0.184	0.686	0.696	0.918	0.310
LASSO-selected	SMOTE	XGBoost	0.771 (0.734–0.807)	0.162	0.764	0.596	0.903	0.339
LASSO-selected	SMOTE	LightGBM	0.764 (0.726–0.801)	0.150	0.834	0.468	0.886	0.364
LASSO-selected	SMOTE	AdaBoost	0.759 (0.725–0.796)	0.213	0.723	0.626	0.905	0.314
LASSO-selected	SMOTE	SVC	0.740 (0.703–0.780)	0.185	0.724	0.655	0.912	0.325
LASSO-selected	SMOTE	MLP	0.725 (0.684–0.766)	0.198	0.706	0.614	0.900	0.297
LASSO-selected	SMOTE	Gaussian NB	0.741 (0.705–0.778)	0.260	0.636	0.702	0.913	0.281
Full candidate set	SMOTE	Logistic Regression	0.753 (0.716–0.793)	0.125	0.962	0.234	0.861	0.556
Full candidate set	SMOTE	Random Forest	0.759 (0.725–0.795)	0.176	0.762	0.550	0.893	0.319
Full candidate set	SMOTE	XGBoost	0.753 (0.714–0.792)	0.147	0.854	0.444	0.884	0.382
Full candidate set	SMOTE	LightGBM	0.744 (0.705–0.784)	0.137	0.897	0.310	0.865	0.379
Full candidate set	SMOTE	AdaBoost	0.756 (0.719–0.792)	0.210	0.845	0.439	0.881	0.364
Full candidate set	SMOTE	SVC	0.728 (0.686–0.769)	0.130	0.957	0.211	0.857	0.500
Full candidate set	SMOTE	MLP	0.706 (0.660–0.747)	0.132	0.976	0.094	0.842	0.444
Full candidate set	SMOTE	Gaussian NB	0.534 (0.508–0.564)	0.172	0.987	0.035	0.835	0.353
LASSO-selected	No SMOTE	Logistic Regression	0.774 (0.734–0.814)	0.122	0.979	0.146	0.850	0.581
LASSO-selected	No SMOTE	Random Forest	0.789 (0.751–0.824)	0.119	0.993	0.082	0.842	0.700
LASSO-selected	No SMOTE	XGBoost	0.781 (0.744–0.817)	0.121	0.970	0.199	0.857	0.576
LASSO-selected	No SMOTE	LightGBM	0.748 (0.703–0.790)	0.124	0.962	0.193	0.855	0.508
LASSO-selected	No SMOTE	AdaBoost	0.791 (0.754–0.826)	0.168	0.987	0.140	0.850	0.686
LASSO-selected	No SMOTE	SVC	0.716 (0.673–0.758)	0.130	0.983	0.105	0.844	0.562
LASSO-selected	No SMOTE	MLP	0.710 (0.667–0.751)	0.128	0.993	0.041	0.836	0.538
LASSO-selected	No SMOTE	Gaussian NB	0.765 (0.730–0.797)	0.163	0.873	0.392	0.876	0.385
Full candidate set	No SMOTE	Logistic Regression	0.768 (0.729–0.806)	0.130	0.955	0.251	0.863	0.531
Full candidate set	No SMOTE	Random Forest	0.783 (0.744–0.819)	0.122	0.995	0.018	0.833	0.429
Full candidate set	No SMOTE	XGBoost	0.784 (0.749–0.819)	0.121	0.968	0.199	0.856	0.557
Full candidate set	No SMOTE	LightGBM	0.764 (0.722–0.804)	0.121	0.976	0.228	0.862	0.661
Full candidate set	No SMOTE	AdaBoost	0.785 (0.750–0.821)	0.175	0.980	0.205	0.859	0.673
Full candidate set	No SMOTE	SVC	0.690 (0.647–0.734)	0.131	0.994	0.082	0.842	0.737
Full candidate set	No SMOTE	MLP	0.659 (0.613–0.701)	0.167	0.903	0.269	0.859	0.359
Full candidate set	No SMOTE	Gaussian NB	0.678 (0.634–0.724)	0.168	0.981	0.053	0.836	0.360

Classification metrics use a probability threshold of 0.50. Calibration and DCA for all 32 configurations are reported in Supplementary Table S4.

### Full-feature and no-SMOTE sensitivity analyses

Using all 36 features did not produce a consistent discrimination advantage for nonlinear models (Figure 3B; Supplementary Table S4). With SMOTE, the full-feature AUC changes relative to LASSO-selected models were −0.003 for logistic regression, −0.005 for random forest, −0.018 for XGBoost, −0.021 for LightGBM, and −0.003 for AdaBoost. Full-feature SMOTE logistic regression had similar AUC but better calibration than LASSO-plus-SMOTE logistic regression (Brier score, 0.125 versus 0.180).

Omitting SMOTE frequently improved discrimination and probability accuracy. With LASSO-selected features, no-SMOTE AUCs were 0.791 for AdaBoost, 0.789 for random forest, 0.781 for XGBoost, and 0.774 for logistic regression. The logistic-regression Brier score improved from 0.180 with SMOTE to 0.122 without SMOTE.

### Calibration and recalibration

The original SMOTE-trained logistic model underestimated the observed delay risk (Figure 3C). Calibration-in-the-large was 1.101, the calibration intercept was 1.377, the slope was 0.793, and the Brier score was 0.180. Logistic regression without SMOTE had calibration-in-the-large 0.276, intercept 0.103, slope 1.197, and Brier score 0.122. Post-hoc logistic recalibration reduced the 10-fold cross-fitted Brier score to 0.123 and calibration-in-the-large to 0.002. Because recalibration used Hospital 2 outcomes, it requires confirmation in a third cohort.

### Decision-curve analysis

The original SMOTE-trained logistic model exceeded both review-all and review-none strategies only at thresholds approximately 0.77–0.95; the no-SMOTE logistic model did so at approximately 0.40–0.95 (Figure 3D). At low thresholds, the high prevalence of delay favored reviewing all records. These curves concern retrospective selection of records for quality-improvement review, not treatment decisions.

### Subgroup absolute risks

In Hospital 2, observed delay risk was 90.5% in rural versus 74.6% in urban residents (absolute difference, 15.8 percentage points; 95% CI, 10.9–20.3). Absolute differences were 21.5 points (95% CI, 18.4–24.5) for non-emergency versus emergency-channel presentation, 9.2 points (95% CI, 4.4–13.6) for referral versus direct presentation, and 36.3 points (95% CI, 23.8–48.7) for non-ambulance versus ambulance transport (Table 4).

**Table 4:** Observed subgroup absolute risks in Hospital 2.

Factor	Exposed, *n*/delayed	Risk, %	Reference, *n*/delayed	Risk, %	Absolute risk difference, percentage points (95% CI)	Risk ratio (95% CI)
Rural residence	546/494	90.5	469/350	74.6	15.8 (10.9–20.3)	1.21 (1.14–1.28)
Non-emergency channel	241/240	99.6	774/604	78.0	21.5 (18.4–24.5)	1.28 (1.23–1.32)
Inter-facility referral	226/204	90.3	789/640	81.1	9.2 (4.4–13.6)	1.11 (1.05–1.17)
Non-ambulance transport	948/811	85.5	67/33	49.3	36.3 (23.8–48.7)	1.74 (1.39–2.30)

Estimates are descriptive observed associations and should not be interpreted as causal effects.

### Model interpretability

**Figure 4:**
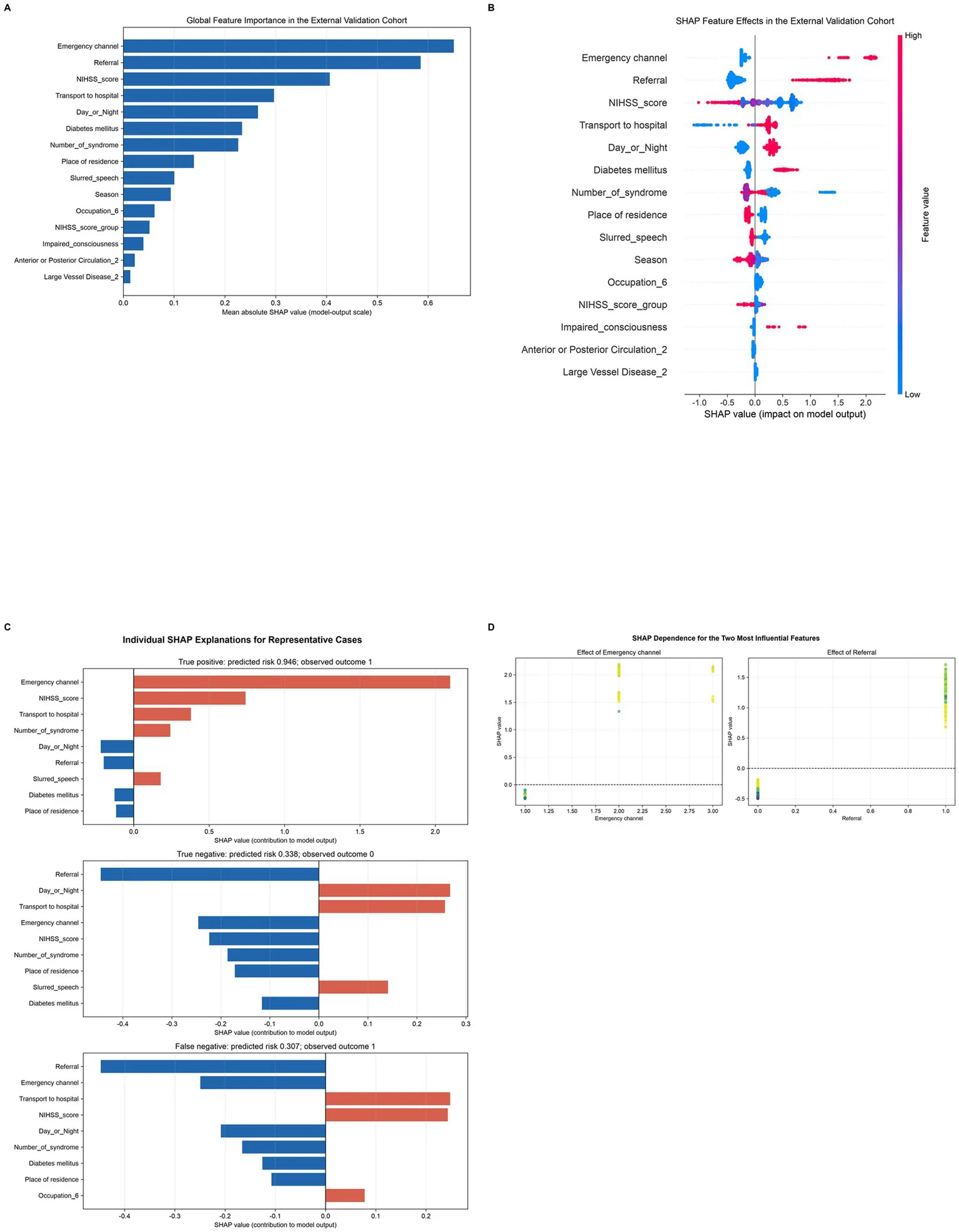
SHAP interpretation of XGBoost, which had the highest external AUC in the prespecified LASSO-plus-SMOTE pipeline, in Hospital 2 **(A)** Global mean absolute SHAP importance. **(B)** Beeswarm feature-effect distribution. **(C)** Separate individual SHAP contribution plots for a true-positive, true-negative, and false-negative case, with predicted risk and observed outcome. **(D)** Dependence plots for emergency-channel presentation and referral, the two highest-ranked features. Mean absolute SHAP values are unitless average magnitudes of contribution on the model-output scale.

SHAP analysis was performed for XGBoost, which had the highest AUC in the prespecified pipeline (Figure 4). Mean absolute SHAP values are unitless magnitudes of average contribution on the model-output scale; they are not probabilities, odds ratios, or causal effects. The individual case plots were reformatted with separated panels and non-overlapping labels.

## Discussion

In this two-hospital retrospective cohort study, we externally validated eight algorithms for prehospital delay in AIS. XGBoost had the highest AUC in the prespecified LASSO-plus-SMOTE analysis, but its difference from logistic regression was small and not statistically significant. Full-feature analyses did not reveal nonlinear gains masked by LASSO selection, and omitting SMOTE generally improved calibration and often discrimination. The principal contribution is therefore the external validation and interpretation of care-pathway factors associated with delay, rather than evidence that complex machine-learning algorithms are superior.

### Comparison with previous studies

Previous studies have examined prehospital delay and related acute stroke care processes (7–9, 26–28). One recent prediction model used internal validation, which may overestimate performance (9). External validation can reveal lower discrimination than development studies, underscoring the importance of evaluation in an independent setting (11, 12). In the present study, the small differences among algorithms did not support a claim that complex models outperform logistic regression.

Our findings align with previous studies identifying rural residence (29, 30), non-ambulance transport (31, 32), and inter-facility referral (33) as factors associated with prehospital delay. We extend this literature by evaluating these factors in an independent hospital and by reporting subgroup absolute risks. Importantly, treatment-related variables were excluded to avoid reverse causality. The low VIFs and negligible correlation between referral and non-emergency-channel presentation reduce concern that these associations reflect multicollinearity, although the estimates remain observational rather than causal.

### External validation and model transportability

A key strength of our study is the use of validation in an independent hospital. The non-random split by setting corresponds to TRIPOD Type 2b (13). The internal out-of-fold XGBoost AUC was 0.776 (95% CI, 0.746–0.807), and the external estimate of 0.771 lay within this interval. This finding supports limited transportability between the two participating hospitals, not broader geographic generalizability.

Calibration was suboptimal in the original SMOTE-trained logistic model, which underestimated the high observed risk. Although restricting SMOTE to training folds prevented leakage, oversampling changed the effective training prevalence and affected probability estimation. No-SMOTE models were better calibrated. Post-hoc recalibration improved probability agreement, but it was exploratory because it used Hospital 2 outcomes and requires validation in another cohort (34).

### Clinical implications

Our analysis identifies several modifiable system-level factors that can inform quality-improvement priorities. Patients presenting through non-emergency channels had substantially higher observed delay risk, supporting public education that emphasizes activation of emergency services for stroke symptoms (35). Referred patients also had higher delay risk, highlighting opportunities to streamline transfer pathways and direct suspected stroke to capable centers (36). Rural residence and non-ambulance transport were associated with large absolute risk differences, supporting improved EMS access, telemedicine-enabled referral support, and targeted education in rural communities (37–39).

Because more than 80% of patients experienced delay, universal measures—multilingual stroke education, promotion of EMS activation, and streamlined referral protocols—remain appropriate. A model should not be used to withhold these measures. Its narrower potential value is to describe pathways with especially high observed risk when intensive resources are limited. The 36.3-point absolute difference associated with non-ambulance transport and the 21.5-point difference associated with non-emergency-channel presentation may help prioritize service review, EMS planning, and targeted education.

It is important to emphasize that several predictors, including NIHSS and stroke-characteristic variables, are recorded only after arrival. The framework cannot predict a delay before it occurs and should not be used for emergency dispatch, real-time triage, or individual bedside prognostication. The decision curves therefore represent retrospective selection of records for detailed quality-improvement review, not treatment of individual patients.

### Model interpretability and clinical adoption

A major barrier to clinical adoption of machine-learning models is difficulty understanding how predictions are generated (40). SHAP analysis provides global and individual descriptions of model output. Mean absolute SHAP values are unitless average magnitudes of contribution on the model-output scale; they are not probabilities, odds ratios, or causal effects. Given the similar discrimination of complex and logistic models, transparent regression estimates and observed subgroup risks may be more useful for health-system communication than individual algorithmic explanations.

### Methodological considerations

All preprocessing parameters were fitted exclusively on Hospital 1 and then applied to Hospital 2, preventing information leakage (41). SMOTE was restricted to training folds, never applied to validation folds or Hospital 2 (42). Feature selection and tuning were also restricted to Hospital 1. To address the concern that logistic LASSO selection could favor additive models, we repeated all eight algorithms with the full feature set and calculated permutation importance for nonlinear models. Full-feature modeling did not reveal an incremental discrimination advantage for nonlinear algorithms. The no-SMOTE analyses showed that correct within-fold implementation prevented leakage but did not prevent altered class prevalence from affecting calibration.

### Limitations

Several limitations warrant consideration. First, both hospitals are located in Kashgar Prefecture, which limits generalizability to regions with different healthcare systems, stroke awareness, and socioeconomic conditions. Validation in other geographic settings is needed.

Second, the high delay rates in both hospitals limit NPV and generalizability to settings with better prehospital systems. XGBoost NPV was 0.339 and the original SMOTE logistic NPV was 0.329. Thus, a low model score cannot reliably identify patients who will not experience delay, and universal interventions must not be withheld from a purported low-risk group.

Third, the framework requires information available only after hospital arrival, precluding individual pre-arrival prediction or real-time triage. It is limited to retrospective health-system profiling and quality-improvement planning.

Fourth, wake-up stroke patients were excluded because symptom-onset time could not be determined reliably. Fifth, potentially important variables were unavailable, including distance to hospital, socioeconomic status, health literacy, household decision-making, language concordance, and initial symptom recognition. Sixth, retrospective registry data may contain recording errors. Finally, post-hoc recalibration used validation outcomes and requires independent confirmation; subgroup differences are associational rather than causal, and small differences among the many model configurations should be interpreted cautiously.

### Future directions

Prospective validation in other geographic and healthcare settings is needed. Implementation studies should evaluate whether targeted education, EMS optimization, and referral-pathway redesign reduce delay. Future pre-arrival models would need variables available to emergency dispatchers, such as caller-reported symptoms, location, and time of day. Qualitative studies may identify additional barriers not captured in registry data.

## Conclusion

Prehospital delay affected more than four in five patients at both Kashgar hospitals. Complex algorithms did not provide a clear advantage over logistic regression, and full-feature analyses did not reveal nonlinear gains masked by LASSO selection. SMOTE worsened probability calibration and was unnecessary for useful discrimination. The framework should be used only for retrospective health-system profiling. Universal quality-improvement measures remain indicated, while subgroup differences in transport mode, emergency-channel access, rural residence, and referral may help prioritize intensive interventions. Prospective validation in another setting is required before probability-based implementation.

## Data Availability

Deidentified data and analysis code will be made available upon reasonable request to the corresponding author, subject to institutional review board approval and data-sharing agreements.’ Patient-level data are not fully contained in the article or Supplementary Material.

## Supplementary material

SUPPLEMENTARY TABLE S1A Missing-data analysis.

SUPPLEMENTARY TABLE S1B Participant flow.

SUPPLEMENTARY TABLE S2 Hyperparameter search spaces and selected values.

SUPPLEMENTARY TABLE S3 External calibration and recalibration.

SUPPLEMENTARY TABLE S4A Calibration and DCA across all 32 configurations.

SUPPLEMENTARY TABLE S4B Paired AUC comparisons.

SUPPLEMENTARY TABLE S4C DCA interpretation.

SUPPLEMENTARY TABLE S5A Six-factor logistic regression.

SUPPLEMENTARY TABLE S5B Variance inflation factors.

SUPPLEMENTARY TABLE S5C Spearman correlations.

SUPPLEMENTARY TABLE S6 Model-specific permutation importance.

SUPPLEMENTARY TABLE S7 Pairwise external AUC comparisons.

SUPPLEMENTARY TABLE S8 Apparent logistic recalibration.

SUPPLEMENTARY TABLE S9 Decision-curve threshold summaries.

SUPPLEMENTARY TABLE S10 Mean absolute SHAP importance.

SUPPLEMENTARY FIGURE S1 ODT distribution by hospital.
