# Supplementary material for: External validation of machine learning models for predicting prehospital delay in acute ischemic stroke: a retrospective cohort study
Source: Front Neurol. 2026 Sep 10;17:1867290. doi: 10.3389/fneur.2026.1867290 (PMC13604024; doi:10.3389/fneur.2026.1867290)
Supplement: Supplementary file 1 [file Image_1.PDF]

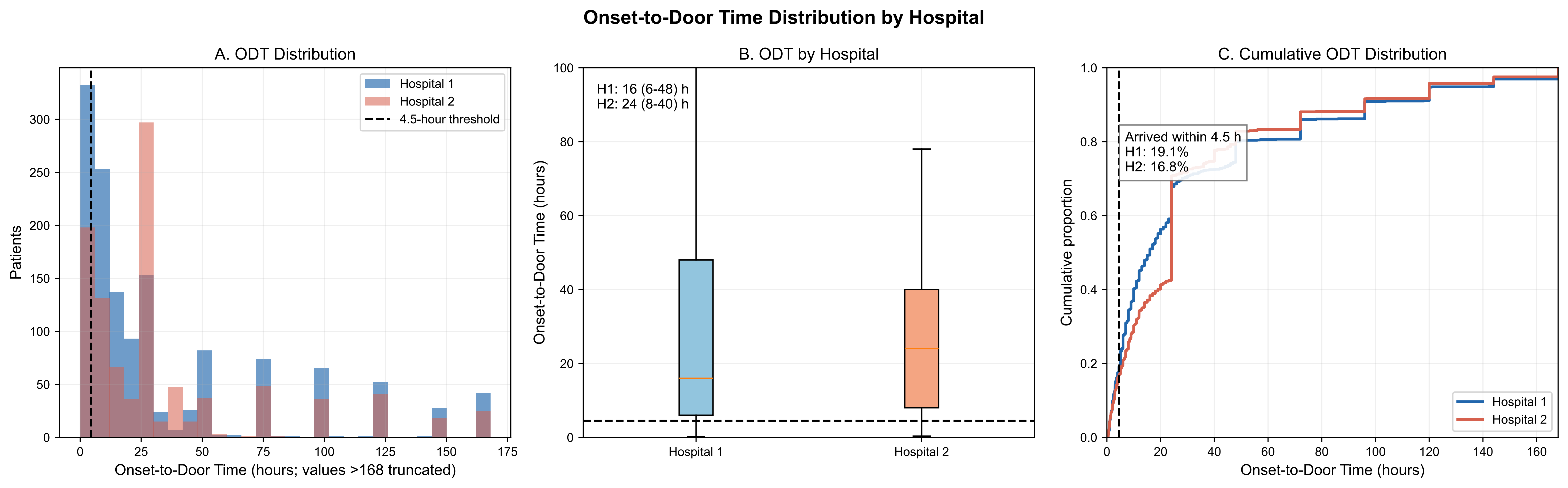
Onset-to-Door Time Distribution by Hospital
A. ODT Distribution
Hospital 1
Hospital 2
4.5-hour threshold
Patients
Onset-to-Door Time (hours; values >168 truncated)
B. ODT by Hospital
H1: 16 (6-48) h
H2: 24 (8-40) h
Onset-to-Door Time (hours)
Hospital 1
Hospital 2
C. Cumulative ODT Distribution
Arrived within 4.5 h
H1: 19.1%
H2: 16.8%
Cumulative proportion
Onset-to-Door Time (hours)
Hospital 1
Hospital 2
